# Understanding the Molecular Basis of Miller–Dieker Syndrome

**DOI:** 10.3390/ijms26157375

**Published:** 2025-07-30

**Authors:** Gowthami Mahendran, Jessica A. Brown

**Affiliations:** Department of Chemistry and Biochemistry, University of Notre Dame, Notre Dame, IN 46556, USA

**Keywords:** rare disease, MDS, lissencephaly, gene expression, therapeutics

## Abstract

Miller–Dieker Syndrome (MDS) is a rare neurodevelopmental disorder caused by a heterozygous deletion of approximately 26 genes within the MDS locus of human chromosome 17. MDS, which affects 1 in 100,000 babies, can lead to a range of phenotypes, including lissencephaly, severe neurological defects, distinctive facial abnormalities, cognitive impairments, seizures, growth retardation, and congenital heart and liver abnormalities. One hallmark feature of MDS is an unusually smooth brain surface due to abnormal neuronal migration during early brain development. Several genes located within the MDS locus have been implicated in the pathogenesis of MDS, including *PAFAH1B1*, *YWHAE*, *CRK*, and *METTL16*. These genes play a role in the molecular and cellular pathways that are vital for neuronal migration, the proper development of the cerebral cortex, and protein translation in MDS. Improved model systems, such as MDS patient-derived organoids and multi-omics analyses indicate that WNT/β-catenin signaling, calcium signaling, *S*-adenosyl methionine (SAM) homeostasis, mammalian target of rapamycin (mTOR) signaling, Janus kinase/signal transducer and activator of transcription (JAK/STAT) signaling, and others are dysfunctional in MDS. This review of MDS integrates details at the clinical level alongside newly emerging details at the molecular and cellular levels, which may inform the development of novel therapeutic strategies for MDS.

## 1. Introduction

The human brain is a complex network composed of billions of neuronal cells, and the intricate organization of this network relies on the precise coordination of various neuronal developmental processes such as neurogenesis, neuronal migration, synaptogenesis, and synaptic pruning [1]. A comprehensive understanding of neuronal development, with a focus on forebrain organization, has been established using various mammalian model systems (e.g., in vitro patient-derived cell lines [2], induced pluripotent stem cells [3,4], human organoids [5,6,7], and rodent models [8,9]), which indicate that alterations in neuronal migration can cause cortical malformations. Neuronal migration, guided by a precise spatio-temporal pattern, is crucial for the establishment of functional neuronal circuits. Disturbances in neuronal circuits can lead to various neurological disorders, such as lissencephaly spectrum disorders, which involve abnormal brain development, including smooth or underdeveloped cerebral cortices [10]. Classical lissencephaly (LIS), characterized by a smooth brain [11,12,13], results from mutations and microdeletions in the *ARX* gene on Chr X [14] as well as from neuronal migration defects caused by mutations and/or microdeletions in the *DCX* gene on Chr X [15,16], *RELN* gene on Chr 7 [17], *PIDD1* on Chr 11 [18], and chromosome 17p13.3. The 17p13.3-derived microdeletions causing LIS can be classified into grades 1–4 based on the severity of the brain’s developmental abnormalities. Grade 1 is the most severe form of LIS, characterized by a smooth brain [19] (complete agyria: absence of gyri (or ridge) on the surface of cerebral cortex and pachygyria: absence of sulci (or indentations) on the surface of the brain that separate the gyri), facial deformities [20,21,22,23,24,25,26,27,28], intellectual disabilities [29,30,31], etc., and is known as Miller–Dieker Syndrome (MDS). The less severe forms of LIS are grades 2–4 and are referred to as isolated lissencephaly (ILS), characterized by smooth brain features (albeit varying degrees of mixed agyria/pachygyria or solely pachygyria) but no observed facial abnormalities [11]. Despite MDS being a distinct condition, it shares many fundamental characteristics with other forms of lissencephaly, particularly the disruption of normal cortical folding and neuronal arrangement. Like other lissencephaly spectrum disorders, MDS presents with developmental delays [13], intellectual disabilities [13], and seizures [32]. The genetic mechanisms underlying MDS, particularly its effects on neuronal migration, are closely related to those seen in other lissencephaly conditions, making MDS a representative model for understanding the broader spectrum of lissencephaly-related disorders and offering potential insights into therapeutic approaches that could be applied across the spectrum.

## 2. Overview of Miller–Dieker Syndrome

**Figure 1:**
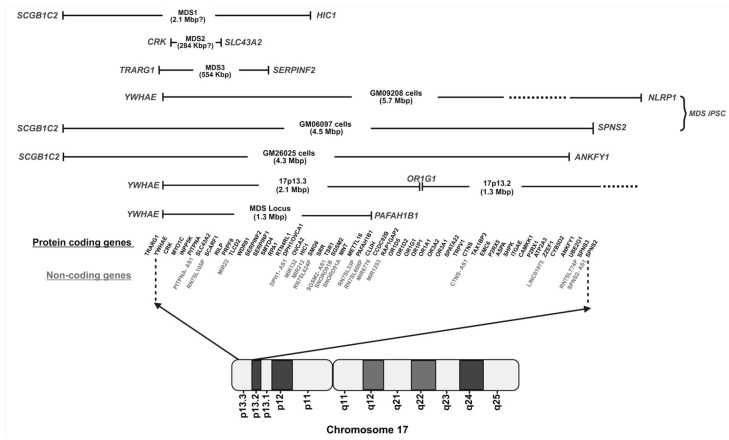
Schematic illustration of MDS-related genes on chromosome 17. Names of all protein-coding (black) and select non-coding (gray) genes in the MDS locus and neighboring regions of human chromosome 17p13.3 are shown. Gene order was obtained from the Human Genome Project Ensemble Database and UCSC Genome Browser [53,54]. Please note that 50 “ENSG” non-coding RNAs were omitted for brevity. Deletions specific to each human-derived MDS cell line (GM09208, GM06097, and GM26025) and reported samples from MDS patients (arbitrarily labeled as MDS 1–3) are denoted by lines with gene names at the boundaries. Base pairs with a question mark denote the uncertainty of the exact deletion. The dotted lines denote additional sequence not shown for spatial reasons. The 1-Mbp region from the telomeric end to the MDS locus (i.e., genes spanning from *SCGB1C2* to *TRARG1*) in 17p13.3 is not shown. Schematic is not drawn to scale. Figure was created using BioRender [55].

MDS (OMIM 247200), named after the two scientists, James Q. Miller and Hillard Dieker, who independently characterized MDS in the 1960s [12,13], is an extreme form of LIS caused by a large heterozygous deletion of 26 protein-coding genes within the MDS locus (i.e., human chromosome 17p13.3 region) that results most notably in brain and facial dysmorphisms (Figure 1). As a rare brain disease, MDS has received limited attention due to its low prevalence: 1 in 100,000 births and fewer than 50,000 people with MDS in the United States [33]. MDS patients often die in utero at 10–20 weeks post-gestation, but children who survive commonly display LIS, developmental delay due to postnatal growth retardation [34,35], distinctive craniofacial features [36,37,38], congenital heart abnormalities (enhanced hypertrophy, ventricular septal defects) [39], ventriculomegaly (large ventricles) [40,41,42], low muscle tone [32,43,44], motor coordination impairment [45,46], bitemporal hallowing [44], scoliosis (sideways curve of the spine) [47], and severe neurological abnormalities resulting in intellectual disabilities [25], seizures [46,48,49], epilepsy [46,48], and reduced lifespan [31]. Besides these issues, MDS patients often undergo surgeries to improve swallowing and breathing difficulties, such as percutaneous gastrostomy and laryngotracheal separation [50], which predisposes them to post-operative seizures. Seizures further increase their risk of inhaling food, liquids, saliva, or vomit into the lungs during or after a seizure, introducing bacteria into the lungs and leading to aspiration pneumonia, which may cause inflammation, breathing difficulties, and severe complications like respiratory failure [39]. Most children with MDS do not live beyond the age of 2 years, and only a few may survive to 10 years. Typically, overall life expectancy is linked to the severity of LIS. As MDS patients grow older, their neurological conditions significantly impact their functional abilities. Therefore, the early detection and management of MDS is crucial. MDS patients can reach adulthood if they receive bracing or surgery for scoliosis [47], if infections due to prolonged hospitalizations are prevented, if antiepileptics are provided for better seizure control, and if the placement of feeding tubes is gentle for individuals with impaired swallowing mechanisms or hypotonia.

## 3. Genetic Basis and Diagnosis of MDS

As chromosome 17 is the second highest in gene density and the third highest in density of segmental duplications [31], human chromosome 17p13.3 is a genomically unstable region that is linked to MDS and other neurodevelopmental diseases, including epilepsy [34]. Thus, this unstable region may give rise to MDS via multiple genetic routes: a ring chromosome (r17) as a result of the fusion of short (p) and long (q) arms of Chr 17 [20], the partial monosomy of 17p13 [20], microdeletions and microduplications in the most distal sub-band of the chromosome 17p arm region [11], contiguous and non-contiguous submicroscopic deletions on 17p13.3 [43], or the microdeletions, resulting in loss-of-function of any 17p gene [51] (Table A1). In general, the microdeletions of MDS patients typically affect a ~3.4-Mega base pair (Mbp) region that covers the entire 17p13.3 region and part of the 17p13.2 region: from gene *YWHAE* to *ANKFY1* (Figure 1). The exact submicroscopic deletions within chromosome 17p13.3 of MDS patients can be determined using polymerase chain reaction (PCR), microarray-based comparative genomic hybridization analysis (aCGH) [27], and fluorescence in situ hybridization (FISH) [52]. Unless otherwise specified in the case of knockout studies, all gene deletions described herein refer to a heterozygous gene deletion (+/−).

A conclusive diagnosis of MDS can be obtained through prenatal chromosomal analysis, such as amniocentesis or chorionic villus sampling, as early as 10–12 weeks, or a fetal brain scan [56]. An ultrasound performed between 18 and 20 weeks of pregnancy can also reveal features suggestive of LIS, such as abnormalities in brain structure [57], which can prompt further genetic testing for confirmation. Future improvements in diagnosing MDS could involve advanced genetic testing such as whole-genome sequencing or chromosomal microarray to identify additional mutations beyond the typical deletions on chromosome 17p13.3 [58]. Integrating genomic data with detailed clinical phenotyping would enhance diagnostic accuracy and allow for a therapeutic plan (see Section 5) to be developed based upon links between specific genetic variants and phenotypic features (see Section 4 below).

More importantly, the timely diagnosis of MDS is critical as it impacts multiple facets of patient management and family support. Clinically, the diagnosis clarifies the cause of severe neurological deficits, notably lissencephaly, which results in significant developmental delays, seizures, feeding difficulties, and often a reduced life expectancy. Establishing a definitive diagnosis enables targeted genetic counseling, helping families understand the genetic basis of the condition, evaluate recurrence risks for future pregnancies, and consider reproductive options such as prenatal testing or preimplantation genetic diagnosis [59]. Moreover, distinguishing MDS from other forms of LIS or related cortical malformations is vital, as these conditions vary in genetic causes, clinical progression, and management strategies. Accurate diagnosis helps avoid unnecessary investigations and guides clinicians toward appropriate, condition-specific care.

## 4. Understanding the Molecular Pathways Underlying MDS Phenotypes

**Figure 2:**
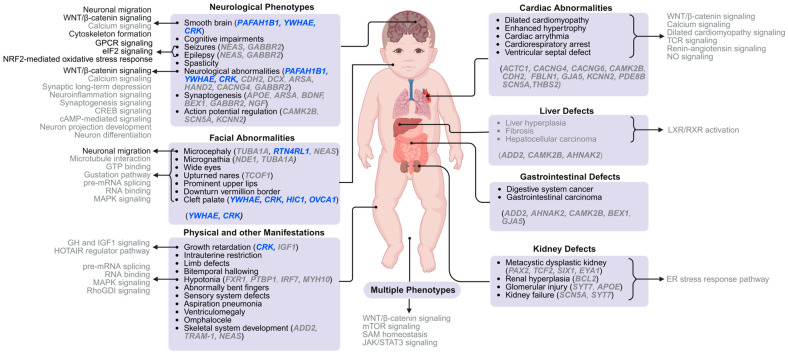
Graphical summary of MDS phenotypes and their associated molecular pathways and genes. Common manifestations of MDS phenotypes are clustered as nervous system defects [4,18,60,61,62,63,64,65], facial dysmorphic features [20,23,24,25,27,37,62,64,66,67], physical and other manifestations [35,38,49,62,64], cardiac abnormalities [39,61,62], liver defects [62], gastrointestinal defects [62,68], and kidney defects [39,62]. Gene names associated with each specific phenotype based on clinical studies are listed in parentheses, and the related molecular pathways, indicated with an arrow from an appropriate bullet point, are on the left or right side. Curly brackets indicate molecular pathways applying to all the covered bullet points. Parenthetical gene names at the bottom of purple box apply to all bullet points. Genes that are encoded in the MDS locus (see Figure 1) are in blue font, while genes in gray are predicted to be associated with each phenotype based on pathway analyses using differentially expressed genes determined by a multi-omics study [61]. Figure was created using BioRender [55].

Our knowledge of different MDS phenotypes stems from the clinical case studies (Table A1), whereby the most prevalent MDS characteristics are (i) LIS due to abnormal neuronal migration and (ii) craniofacial dysmorphic features. Early studies primarily focused on two pivotal genes in the MDS locus (Figure 1): *PAFAH1B1* (also known as *LIS1*) and *YWHAE* (also known as *14-3-3ε*) [11,29]. To fully understand the molecular basis of the aforementioned characteristics along with abnormalities impacting cardiac function, organ development, and motor regulation, it is essential to create a comprehensive map of MDS phenotype-related genes and pathways, which will enable a more effective long-term therapeutic approach tailored to each MDS patient. Figure 2 displays the common clinical phenotypes or abnormalities of MDS patients alongside their associated gene candidates and their relevant pathways.

Creating this network relied upon reported cytogenetic analyses of MDS patient cells [38,43,69,70,71,72,73], improved model systems for MDS (e.g., organoids [4,18,74,75] and in vivo rodent knockout studies [65,66,76]), and multi-omics analyses [61,74,75] to identify MDS-associated differentially expressed genes (DEGs). Recent breakthroughs in model organoids have enabled a more precise mimicry of human tissues, making organoids powerful tools for drug testing, disease modeling, and the development of personalized medicine. In addition, “omics” studies performed on human MDS organoids and patient-derived MDS cells, namely transcriptomics (RNA-seq [61] and single-cell RNA-seq (scRNA-seq) [4,18,74]) and proteomics (mass spectrometry [18,61]), whose gene lists were analyzed using bioinformatic tools (Gene Ontology, QIAGEN Ingenuity Pathway Analysis, and Kyoto Encyclopedia of Genes and Genomes) have generated many predictions that require further examination to establish if they are *bona fide* contributors to MDS. It is important to note that a major challenge in establishing clear genotype–phenotype relationships is that multiple genes within the 17p13.3 region (Figure 1) are deleted and that microdeletions are unique to each MDS patient (Table A1), highlighting the need to study the effects of single- and multi-gene deletions. For the remainder of the review, we will focus on the molecular basis of the most prominent phenotypic features of MDS: the “smooth brain” condition caused by neuronal migration defects (Section 4.1), distinctive craniofacial features (Section 4.2), and other characteristics (Section 4.3). In addition, there are several notable molecular pathways and mechanisms (Section 4.4) that have been elucidated at the cellular level and may underlie a myriad of phenotypes. We first present the impact of gene deletions within the MDS locus and then highlight the potential roles of DEGs outside this region and have established connections to the related phenotype. The DEGs identified through multi-omics approaches in MDS offer deeper insights into the molecular landscape of MDS [4,61,75].

### 4.1. Smooth Brain

Classical LIS or smooth brain arises from severe neuronal migration defects occurring between 10 and 20 weeks post-gestation [19]. During normal brain development, neurons are guided to their appropriate locations within the cerebral cortex. Neurons form layers as they migrate to their final positions, contributing to the formation of gyri and sulci on the brain’s surface [4]. Gyri and sulci increase the surface area of the brain, allowing for more neurons to migrate and enhance cognitive abilities [77]. For MDS patients, defective neuronal migration leads to the underdeveloped and thickened cerebral cortex that lacks gyri and sulci [18,78]. This smooth brain surface has a significantly reduced surface area, impairing cognitive and motor functions [79]. scRNA-seq studies on MDS patient-derived induced pluripotent stem cell (iPSC)-generated organoids [4] (Figure 1, see GM06097 and GM09208), along with a conditional knockout (CKO) study utilizing *Lis1^hc/ko (or −/−)^* mice (where hc refers to a hypomorphic-conditional allele and ko refers to knockout) and *Ndel^hc/hc (or −/−)^* mice [80], have identified cell migration defects caused by loss of *PAFAH1B1/LIS1* and *YWHAE*, as well as other genes involved in cortical developmental malformations: *ASPM*, *CTIP2, NDEL1 or NDEL, PAX6*, and *SOX2*. The researchers identified multiple cellular defects associated with the loss of LIS, including decreased cell migration, increased apoptosis of neuroepithelial stem cells, and more horizontal cell divisions. A key finding from this study was a mitotic defect in the outer radial glia (oRG) cells, which is crucial for human neocortical expansion but largely absent in lissencephalic rodents [4]. Another recent study [18] supported this finding, demonstrating a decrease in oRG progenitor cells and an increase in horizontal and oblique divisions, which, respectively, enable cell renewal and different cell types; these findings are consistent with severe-grade LIS1-lissencephaly. Importantly, these studies demonstrated the effectiveness of using cerebral organoids to model human neurodevelopmental disorders by examining cell types and processes specific to human cortical development [4,18]. Thus, these organoids recapitulate key features of LIS, including defective neuronal migration based on a wound healing assay and the absence of proper cortical folding based on live-cell imaging, emphasizing the importance of oRG cells in human cortical development [4].

**Figure 3:**
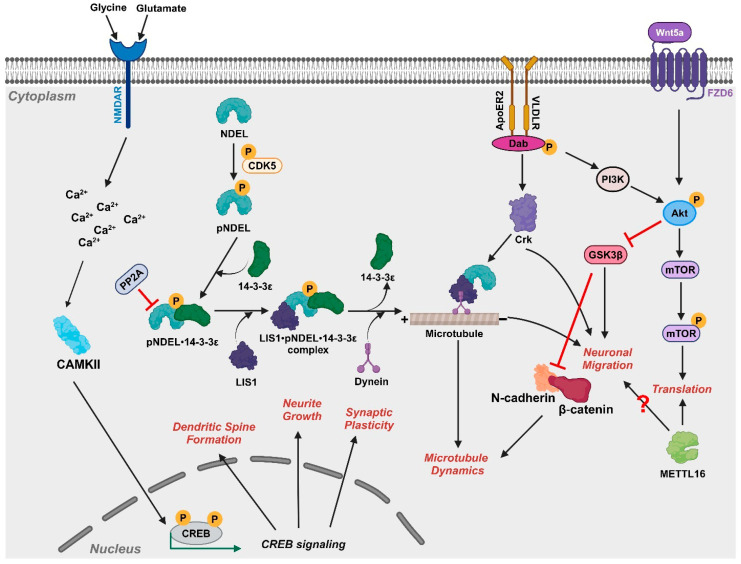
Key gene actions in MDS progression and their role in brain development. Summary of the key genes discovered in MDS progression (*PAFAH1B1/LIS1*, *YWHAE/14-3-3ε*, *NDEL*, *CRK*, *METTL16*, *WNT*), participating in processes such as neuronal migration, microtubule organization, actin cytoskeleton stabilization, and protein translation [18,61,63,89]. Figure was created using BioRender [55].

Mutations or deletion of *PAFAH1B1/LIS1* [81] and *YWHAE/14-3-3*ε [82] (Figure 1) can result in severe LIS as seen in MDS patients [63]. At the molecular level, LIS1 binds directly to cytoplasmic dynein and microtubules, facilitating proper spindle positioning and neuronal migration, while also playing a dual role in orchestrating both microtubules and the actin cytoskeleton by interacting with tubulin to stabilize microtubule dynamics [81] (Figure 3). These cytoplasmic dynein-mediated processes of LIS1 include cell motility, nucleokinesis [83], and mitotic somal translocation associated with neurogenesis and chromosomal segregation [84]. As a remarkably conserved protein, 14-3-3ε interacts with and safeguards phosphorylated NDEL (pNDEL), preventing its dephosphorylation by protein phosphatase 2A (PP2A) [85] (Figure 3). LIS1 and pNDEL mainly co-localize at the centrosome in early neuroblasts but relocate to axons alongside retrograde dynein motor proteins [86]. The co-localization of the LIS1•pNDEL•14-3-3ε complex is essential for regulating neuronal migration and centrosome activity during early neurogenesis [81]. Based on independent studies conducted using single-gene KO mice of *PAFAH1B1* (*Lis1* KO *^(or −/−)^*) [87], *YWHAE* (*Ywhae^−/−^*) [88], and *CRK* (*Crk^FL/FL (or −/−)^: where FL refers to floxed*) [89], 14-3-3ε plays a critical role in guiding phosphorylated NDEL1 (pNDEL1) to specific cellular regions where it can interact with the molecular machinery responsible for intracellular transport. By stabilizing this localization, 14-3-3ε ensures the proper function of cytoplasmic dynein—a motor protein that travels along microtubules—thereby supporting the directional movement of neurons during brain development and contributing fundamentally to the process of neuronal migration [63]. Moreover, MDS-iPSC-derived forebrain-type organoids (Figure 1, see MDS-iPSC) showed that alterations in the microtubule organization of ventricular zone radial glial cells (vRGCs, which are the primary neural stem cells of the developing cortex that form the structural and functional backbone of the ventricular zone (VZ) niche) and the disruption of cortical niche architecture (i.e., a specialized microenvironment in the brain cortex that regulates neural progenitor maintenance, cell division orientation, and neuronal migration) led to an impaired N-cadherin/β-catenin signaling axis [74,75]. Due to the haploinsufficiency of *PAFAH1B1* and *YWHAE* in MDS, neuronal migration defects contribute to the “smaller head” phenotype (i.e., microcephaly) observed in these organoids, primarily through the disruption in the cortical niche architecture [75] (Figure 3). Similarly, MDS patient-derived organoids were significantly smaller, with vRGCs shifting from symmetric (i.e., vRGC divides to produce two identical progenitor cells) to asymmetric cell division (i.e., vRGC divides to produce one progenitor cell and one differentiated cell) [75]. Reinstating active β-catenin signaling returned cells to symmetric division and improved growth defects in the organoids [75]. Similarly, severe LIS1-lissencephaly patient organoids (Grade 1) showed a notable decrease in Wnt signaling, which disrupted the architecture of the ventricular zone (VZ) niche [75]—an essential region in the cortical niche where neural stem cells proliferate and initiate cortical neurogenesis—and subsequently reduced the expression of cell adhesion molecules due to abnormal microtubule dynamics [74]. Thus, this study highlights the roles of *PAFAH1B1* and *YWHAE* in maintaining the cortical niche and demonstrates the utility of organoid-based systems for modeling complex cell–cell interactions in vitro [74]. Another recent study employed a multi-omics approach on patient-derived organoids to model mild, moderate, and severe LIS1-lissencephaly to examine the gradients of LIS-related disease severity [74]. Disruptions in LIS1 markedly dampen Wnt signaling, hindering the proliferation and differentiation of neural progenitor cells during brain development and decreasing neuronal migration and abnormal cortical patterning. These findings emphasize the crucial role of Wnt signaling in proper brain development and highlight its potential as a therapeutic target for addressing the defects underlying lissencephaly.

Interestingly, the reduced expression of METTL16 (methyltransferase-like protein 16), an m^6^A (*N*^6^-methyladenosine) RNA methyltransferase, decreased cell migration of the patient-derived MDS cell line GM06097 (Figure 1) [61], but the overexpression of METTL16 increased cell migration based on a wound healing assay, suggesting that METTL16 may also affect neuronal migration as observed for *PAFAH1B1* [90], *CRK* [67] and *YWHAE* [25] haploinsufficiency. It is not yet known how METTL16 contributes to cell migration in MDS cells as well as cancer [61,91]. Multiple proteins may contribute to defective cell migration in MDS patients, although *PAFAH1B1*, *YWHAE*, and *CRK* genes are the major contributing factors.

### 4.2. Facial Dysmorphic Features

Because the MDS locus 17p13.3 is haploinsufficient in most MDS patients, the genomic imbalances of 26 gene deletions (Figure 1) were assumed to induce critical brain malformations and distinctive facial dysmorphisms, including microcephaly (smaller head) [20], micrognathia (smaller lower mandibles) [92], flattened midface [22], prominent forehead [21], cleft palate [27], laterally extended eyebrows [26], maxillary prominence [93], prominent upper and/or lower lip [28,67], short nose with upturned nares [23,25], low set posteriorly rotated ears [20,28], and downturned vermillion boarder [24] (Figure 2). These distinctive features in MDS patients provide insights into disease severity and associated neurological impairments, helping clinicians to identify the disorder early and provide the early intervention and management of MDS. Additionally, the distinctive features can also indicate the underlying genetic and neurodevelopmental aspects of the syndrome, which may inform prognosis and the need for specialized care.

Of all the reported 17p13.3 microdeletions in MDS patients, one patient had a 2.1-Mbp deletion of the MDS locus involving the haploinsufficiency of *YWHAE*, *CRK, OVCA1,* and *HIC1* but not *PAFAH1B1* (Figure 1, see MDS1), and this patient displayed significant craniofacial dysmorphisms along with other MDS phenotypes [41] (Figure 2). Based on their mutational analysis, *YWHAE* and *CRK* are thought to be implicated in major facial dysmorphic traits arising from a severe neuronal migration defect and a neural crest migration defect, respectively [25,67]. A patient having a 284 kbp deletion in the MDS locus, spanning *CRK* but not *YWHAE* (Figure 1, see MDS2), revealed slight facial defects, suggesting a potential role of CRK in the observed facial phenotypes [67]. In addition, a deletion spanning from the *TRARG1* to *SERPINF2* region, but not *PAFAH1B1*, was associated with a range of defective craniofacial traits, along with growth retardation, cognitive impairments, and brain malformations [25,36,94] (Figure 1, see MDS3). In a mouse model, two other genes within the MDS locus, *HIC1* and *OVCA1*, were previously described to have an association with cleft palate and nasal formation [95], and mandible formation [66], respectively. Although the resulting phenotypic consequences of a single-gene deletion without another have not been investigated systematically, these natural variations in gene deletions highlight the significant impact of certain genes, namely *YWHAE*, *CRK*, and *PAFAH1B1*, on the resulting anatomical features. Apart from the genes within the MDS locus, there are other genes (*TUBA1A*, *NDE1*, and *TCOF1*) whose contribution to these facial characteristics have not been investigated in MDS, but they are known to play a key role in craniofacial phenotypes [76,96,97] (Figure 2). Mutations in TUBA1A disrupt neuronal migration and the cytoskeleton, which can lead to facial deformities such as micrognathia and a broad nasal bridge, traits that are commonly seen in MDS [98,99]. NDE1, which works closely with LIS1, plays a key role in brain development and cell division; when mutated, it can cause microcephaly and additional facial abnormalities, potentially worsening MDS facial features [100]. TCOF1, which is crucial for the survival of neural crest cells, is associated with Treacher Collins syndrome—a condition sharing many craniofacial similarities with MDS, including underdeveloped jaw structures [101]. Interestingly, a gene called *NEAS*, which is also known to have connections to facial features, has been identified as differentially expressed in transcriptomics studies [61]. While the precise function of NEAS is not fully understood, its involvement in neural or neural crest development suggests it could also influence facial structure formation in MDS. Together, these genes are part of essential pathways in craniofacial development, each potentially contributing to the distinct facial characteristics seen in MDS. Thus, increased attention should be given to understanding the expression patterns of the genes that contribute to the facial abnormalities in MDS to enable the development of more effective diagnostic tools, targeted therapies, and personalized interventions to improve both the physical and developmental outcomes.

### 4.3. Other Characteristics of MDS Patients

Besides smooth brain and the prominent facial dysmorphic features, MDS patients also exhibit other phenotypic features, symptoms, and/or complications. For example, generalized epilepsy and intractable seizures are closely associated with LIS, as MDS infants who have more gene deletions often experience more severe forms of epilepsy [41] (Figure 2). GABBR2, encoded by GPCR, is involved in slow inhibitory neurotransmission, and plays a vital role in maintaining excitatory/inhibitory balance. GABBR2 was found to be differentially expressed in MDS cells [61] and mutations of GABBR2 have been identified in various epilepsies, leading to neuronal hyperexcitability and seizures [102]. In disrupted GPCR signaling, impaired GABBR2’s function contributes directly to epilepsy in cortical malformations such as LIS [103]. Beyond neurotransmission, GABBR2 dysfunction also initiates neuronal stress responses, including the activation of the eIF2α phosphorylation pathway, a hallmark of the integrated stress response during seizures [104]. Finally, excessive neuronal activity from loss of GABBR2 enhances oxidative stress, triggering the activation of the NRF2 antioxidant pathway [105]. Adding to this mechanistic framework, the NEAS gene, also known as SCN1A is a well-established epilepsy gene whose mutations are frequently associated with epileptic encephalopathies [106]. Loss-of-function mutations in NEAS impair the excitability of GABAergic interneurons, disrupting inhibitory control and leading to uncontrolled excitatory activity, seizures, and cognitive impairment [107,108]. NEAS dysfunction is also linked to the secondary activation of stress and inflammatory pathways, and its interaction with broader signaling networks, including GPCRs, ISR, and redox mechanisms, further exacerbates seizure susceptibility [108]. Hence, GPCR, eIF2, and NRF2 signaling pathways form an interconnected triad in seizure pathogenesis and signify therapeutic importance. Another problem related to seizures is hypotonia or muscle weakness, stemming from lack of muscle use [28] (Figure 2). While some MDS patients may exhibit early motor development, including proper head control and belly crawling, motor control is often lost after the onset of seizure activity [28].

**Figure 4:**
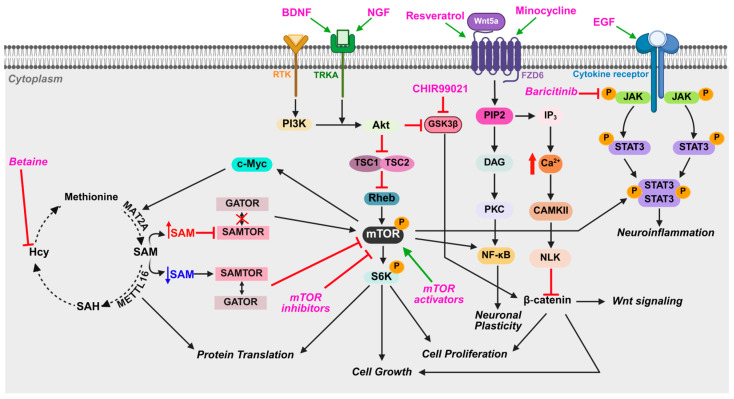
Potential therapeutic targets for treating MDS symptoms. The schematic summarizes the key metabolic pathways, and their associated effectors implicated in MDS. The pathways are shown with key enzymes, receptors (RTK, TRKA, cytokine receptor, FZD6), molecules involved, and their potential effects on cellular processes. Areas where drug intervention (magenta color) could inhibit (red blunt end) or enhance (green arrow) the metabolic activity to improve treatment outcomes are indicated. Figure was created using BioRender [55].

MDS patients experience various compromised organ functionalities: congenital heart anomalies (e.g., patent ductus arteriosus, pulmonary hypertension, atrial septal defect) [28,39]; acute respiratory distress syndrome [39,109], leading to ventilator and feeding tube [93]; chronic gastroesophageal reflux disease [68]; genitourinary anomalies (renal anomaly and cryptorchism) [39]; liver hamartoma (non-cancerous growth made up of normal types of cells and tissues, but they are arranged in a disorganized way) [13], omphalocele (a birth defect where babies are born with some organs sticking out through the belly button) [49], and limb anomalies (contracture and polydactyly) [110] (Figure 2). Consistent with these clinical findings, a recent MDS study identified specific genes and pathways that may explain compromised organ functions, including cardiac hypertrophy (*ACTC1, CACNG4, CACNG6, CAMK2B, CDH2, FBLN1, GJA5, KCCN2, PDE8B, SCN5A, THBS2*, skeletal system development (*ADD2*, *TRAM-1*, *NEAS*), calcium signaling (*CAMKIIB*, *PDE8B*), synaptogenesis (*APOE*, *ARSA, BEX1, GABBR2*), and the STAT3 pathway (*WNT16*) [61]. In addition, these studies also pointed out enhanced calcium signaling, cAMP signaling, and activated CAMKIIB expression in MDS-patient derived cells [61]. Calcium signaling activates CAMKII, which phosphorylates and activates CREB signaling, leading to the transcription of genes like CAMKIIB that encode for CAMKII in a positive feedback loop [111] (Figure 4). CAMKII signaling is involved in calcium-dependent signaling during the early stages of postnatal and mature brain development [111], although its function in neuronal activity remains a question. Calcium in cardiac muscles is regulated by CAMKII, which plays a key role in muscle contraction and cardiac hypertrophy. Hence, the CAMKII overexpression observed in MDS is expected to be one of the primary causes for dysregulated calcium signaling and contribute to activated cardiac hypertrophy signaling [61] (Figure 2).

### 4.4. Notable Molecular Pathways and Mechanisms

As already mentioned, the pathways for WNT/β-catenin signaling and calcium signaling may underlie multiple phenotypes like smooth brain, severe neurological defects, and distinctive facial abnormalities (Figure 2) [61,75]. However, ‘omics’ analyses have identified more molecular pathways in MDS cells/organoids that deviate from normal [61,74]. Several recent studies have highlighted a role for the mTOR pathway in MDS: two indicate mTOR signaling is downregulated [18,61], while one indicates mTOR signaling is upregulated [74]. Using GM06097 cells as a model system for MDS (Figure 1), one study reported that mTOR signaling is reduced due to decreased SAM/SAH ratio and downregulated METTL16 expression, an m^6^A methyltransferase that regulates SAM homeostasis [61] and translation [91,112,113]. A similar finding was observed for MDS cerebral organoids: suppressed protein translation, metabolic disruption, and hypoactive mTOR signaling [18]. Reduced mTOR complex 1 (mTORC1) activity underlies these structural and functional abnormalities [18,61]. Critically, treatment with a brain-selective mTORC1 activator not only prevented but also reversed cortical thickening and restored protein synthesis and metabolic homeostasis [18]. These findings establish mTOR hypoactivity as a shared mechanistic driver in lissencephaly spectrum disorders and highlight mTOR activation as a promising therapeutic strategy [18,61].

However, another recent study reported upregulated mTOR signaling in MDS patient-derived forebrain organoids, revealing a greater degree of activated mTOR signaling in organoids exhibiting a more severe form of LIS [74]. Severe LIS organoids showed pronounced defects in progenitor cell homeostasis, microtubule stability, and cortical organization, which correlated with hyperactive mTOR signaling and downregulated WNT signaling genes. Single-cell transcriptomic analysis revealed altered cell fate decisions tied to mTOR pathway imbalances, contributing to cortical malformation severity [114]. Treating MDS organoids with the mTOR inhibitor everolimus partially rescued these phenotypes, highlighting mTOR signaling as a key mediator of disease progression and a potential therapeutic target in LIS1-lissencephaly.

One possible reason for these conflicting variations in mTOR signaling may be differences in disease severity and developmental timing: while mTOR is hypoactive in MDS organoids and MDS cells due to metabolic and translational deficits and different pathway contributions, it becomes hyperactive in severe LIS1-mutant organoids as a compensatory response to cytoskeletal instability and WNT dysregulation. These results suggest that mTOR dysregulation in LIS is dynamic and context-dependent, shifting from suppressed to overactivated states depending on the underlying genetic insult and stage of neural progenitor cells during cortical development. For example, oRG display notably high levels of mTORC1 activity, followed by protein S6 (pS6) phosphorylation [115]. In contrast, other progenitor types such as vRG, truncated radial glia (tRG), and intermediate progenitors (IPCs) show minimal mTORC1 activation, pointing to a specialized dependence of oRG on this pathway [116,117]. mTORC1 disruption, either via rapamycin treatment or genetic interventions, specifically impairs oRG morphology and migration, suggesting a cell type–specific role for mTOR signaling in regulating structural and migratory features rather than general cell division [115]. In addition, mTOR signaling in neural progenitors is tightly modulated by external metabolic conditions such as mitogenic stimuli (insulin, IGF-1, and FGF2), which can cause mTORC1 activation, whereas nutrient scarcity or hypoxic conditions suppress its activity [118]. These environmental inputs influence not only progenitor cell proliferation and survival but also govern transitions between quiescent and activated states, particularly in adult neural stem cell niches. Overall, these findings highlight the intricately regulated, context-dependent nature of mTOR signaling across progenitor subtypes and environments, underscoring the importance of further research into how these dynamics shape neural lineage decisions and brain development.

Because there is an imbalance in SAM/SAH levels in MDS GM06097 cells (Figure 1), there is the potential that SAM-dependent methylations may be perturbed in DNA, RNA, and protein. Thus far, the global levels of 33 nucleoside modifications, including prominent modifications such as 5mC in DNA as well as m^6^A and pseudouridine in RNA, have been examined and showed no significant changes in GM06097 versus non-MDS cells [61]. However, a comparative analysis of site-specific modifications in DNA, RNA, and protein, particularly m^6^A in RNA, could contribute to MDS phenotypes. For example, m^6^A dysregulation occurs in several neuronal diseases [119,120] and neurodevelopmental disorders, such as autism spectrum disorder [121], intellectual disability disorders [122], fragile X syndrome [120], Alzheimer’s disease [123], and Parkinsons’s disease [124]. Notably, m^6^A marks have essential roles during embryonic stem cell differentiation [125], brain development [126], learning and memory [127], including METTL16-dependent m^6^A marks [127]. m^6^A is the most prevalent mRNA modification in the brain, and reducing METTL16 is known to alter m^6^A marks catalyzed by the major m^6^A mRNA methyltransferase complex, METTL3 and METTL14 [91,128,129,130]. SAM/SAH imbalances may also affect DNA and protein methyltransferases, which regulate protein and histone methylation, respectively. In addition to methylation events, other post-transcriptional and post-translational modifications, such as phosphorylation, should be examined in MDS, as phosphorylation levels vary greatly in the mTOR signaling pathway of MDS cells [18,61]. DNA, RNA, and protein modifications represent a major knowledge gap in our understanding of MDS.

## 5. Current and Potential Treatment Options for MDS

MDS is caused by a de novo mutation; therefore, the efficacy of treatments depends on a timely diagnosis (see Section 3). The early and accurate identification of MDS allows healthcare providers to anticipate challenges, customize treatment plans, and implement early supportive interventions. Currently, the only available treatments are symptom-based and aim to mitigate symptoms and prevent further complications. The major symptoms include recurrent seizures, varying intellectual disabilities, cardiac and renal complications, developmental delays and motor coordination impairments (Figure 2). Seizures are managed using anti-seizure medications (e.g., phenobarbital, valproate, zonisamide, vigabatrin, clobazam, topiramate, levetiracetam), which are typically ineffective due to the development of drug-resistant seizures and place a considerable burden on patients and caregivers. A retrospective cohort study recently showed that the administration of perampanel (PER) [131] was effective at treating drug-resistant seizures in only 50% of MDS cases [132]. Corpus callosotomy is another option that successfully relieves the drug-resistant epileptic spasms [133]. As described in Section 4.3, epilepsy and seizures are related to GPCR signaling, eIF2 signaling, and the NRF2-mediated oxidative stress response; therefore, those pathways also offer potential therapeutic benefits. Baclofen, a GABBR2 agonist, has been shown to restore inhibitory signaling and reduce seizures in animal models, highlighting the therapeutic potential of targeting GABBR2-mediated GPCR pathways [134]. Elevated phospho-eIF2α reduces protein synthesis and synaptic plasticity, exacerbating seizure susceptibility [135]. Targeting this pathway with ISR inhibitors (e.g., ISRIB) has mitigated behavioral and electrophysiological abnormalities in epilepsy models, offering a promising strategy for LIS-related syndromes [136]. Lastly, NRF2 activators (e.g., sulforaphane, dimethyl fumarate) reduce oxidative damage, neuronal loss, and seizure frequency [137]. This supports NRF2-targeted neuroprotection as a complementary strategy in managing epilepsy arising from cortical malformations [138]. In addition to seizure control, managing MDS depends on developmental and physical therapies, feeding tube, regular monitoring for cardiac and renal complications, and behavioral and educational interventions tailored to the individual’s needs. Multidisciplinary care, including genetic counseling and psychosocial support, is also essential.

Therapeutic strategies, including some drugs already approved by the Food and Drug Administration for other conditions, are emerging from a better understanding of the molecular pathways underlying MDS (Figure 2 and Figure 3). For example, the JAK/STAT signaling pathway is a promising target for drug development in other neurodegenerative diseases. Based on one transcriptomics study, activated STAT3 signaling and downregulated IL-1β signaling in MDS-patient derived cells [61] likely contributes to neurological and developmental abnormalities causing impairment in neuronal differentiation, migration, synaptic formation, and neuroinflammation [61]. Therefore, brain-derived neurotrophic factor (BDNF) and JAK inhibitors (JAKi), such as baricitinib and AZD1480 used to decrease neuroinflammation and inhibit STAT3 activation, may be potential therapies to explore because they would alleviate neuroinflammatory responses, reduce neuronal damage, and promote neuroprotective effects in MDS [139,140] (Figure 3 and Figure 4).

Decreased Wnt/β-catenin signaling has been observed in MDS patient-specific forebrain-type organoids (Figure 1, MDS iPSC), which results from the altered microtubule network organization and disruption of cortical niche architecture [75]. Therefore, compounds that enhance the canonical Wnt/β-catenin signaling pathway and Rho-GTPase activity (Resveratrol [141], Minocycline [142]) may aid in promoting neurogenesis and improve cognitive function as they do for diseases like Alzheimer’s [143], Parkinson’s [144], and Huntington’s [145]. Furthermore, exploring the regulation of the SAM/SAH ratio, which is lower in MDS cells, provides another therapeutic avenue (Figure 4) because a lower SAM/SAH ratio, which lowers methylation potential and impairs epigenetic modifications and neurotransmitter synthesis, could contribute to neurological defects, cognitive impairments, seizures, and cardiovascular abnormalities [146,147]. One possible treatment would be methylating agents like betaine, which can help normalize the SAM/SAH ratio by reducing SAH levels [148]. METTL16 is a novel drug target because it is implicated in not only the SAM/SAH imbalance but also cell migration [61].

Interestingly, the mTOR pathway has recently gained significant attention as a convergent point of intervention for LIS pathogenesis. Two recent studies revealed a decrease in mTOR-dependent protein translation (Figure 3) in GM06097 cells and MDS organoids (Figure 1, see GM26025), suggesting mTOR activators as a potential therapeutic [18,61]. This possibility was confirmed by treating MDS organoids with the mTORC1 activator NV-5138, which acts through GATOR (Figure 4). NV-5138 treatment was able to prevent and reverse both cellular and molecular defects observed in MDS by enhancing mTOR signaling [18]. NV-5138 stimulates mTORC1 signaling by targeting Sestrin2, which is an amino acid sensor. The binding of NV-5138 to Sestrin2 displaces the GATOR2 complex, lifting the blockade on mTORC1 activity. NV-5138 crosses the blood–brain barrier and acts specifically in brain regions such as the prefrontal cortex [149]. Together, these results highlight the potential of targeting mTOR signaling as a promising therapeutic strategy.

In contrast, another recent study demonstrated that mTOR pathway inhibitors, such as everolimus, act by forming a complex with the protein FKBP12, which then inhibits the mTORC1 pathway is involved in cell growth and metabolism. This inhibition disrupts signals required for cell division and survival, ultimately slowing tumor growth and proliferation [150]. Hence, everolimus could reverse the phenotypic changes (e.g., neuronal migration defects) observed in organoids derived from lissencephaly spectrum disorders [74]. Therefore, these findings suggest that activated mTOR signaling could contribute to a range of pathologies (e.g., tuberous sclerosis or TSC, focal cortical dysplasia, hemimegaloencephaly, autism, epilepsy, and intellectual disability), highlighting the complexity of mTOR’s role in neurodevelopmental disorders [151]. Although these results contradict earlier research [18,61], showing that mTOR pathway activation leads to the reversal of cellular and molecular defects in lissencephaly organoids, they suggest that mTOR dysregulation may underlie various brain malformations and symptoms with differing degrees of severity, positioning mTOR as a promising target for therapeutic interventions.

In addition, nerve growth factor (NGF) and epidermal growth factor (EGF) could be explored as therapeutic agents to enhance protein translation and cellular function in MDS patients [152] (Figure 4). By correcting the disrupted protein synthesis pathways, these growth factors may offer a means to improve the neurological, cardiovascular, and other systemic defects seen in MDS. However, further research and clinical studies would be needed to evaluate the safety, efficacy, and potential benefits of NGF and EGF treatment for MDS. Additionally, targeting specific gene candidates involved in neuronal processes, such as synaptogenesis (*APOE*, *ARSA*, *BEX1, BDNF*, *NGF*, *GABBR2*), action potential regulation (*CAMKIIB, SCN5A, KCNN2*), and cytoskeleton formation (*ACTG1*, *PAFAH1B1*, *YWHAE*), may offer novel treatments.

In theory, genome editing and gene replacement therapy represent potential therapeutic options for MDS. CRISPR/Cas9 genome editing could be used to directly correct the genetic mutations responsible for defects in key genes, such as *PAFAH1B1*, *YWHAE*, and *CRK*, either by fixing point mutations or inserting functional copies of these genes into the affected areas of the genome [153]. For example, the possible point mutations in MDS-related genes in *PAFAH1B1* include nonsense, missense, and frameshift mutations (e.g., c.164C > T, c.358C > T, c.589delG), which disrupt protein function and contribute to neuronal migration defects [154]. Importantly, there is a precedent for how patient-derived models can be used to tailor gene editing to the individual’s genetic makeup, enhancing the precision and safety of the treatment [155]. By correcting the disease-causing mutations in the patient’s cells, the researchers regained the normal gene function and alleviated disease symptoms [155]. The findings underscore the potential of personalized in vivo gene editing as a transformative tool in treating genetic disorders, paving the way for more effective, targeted therapies in the future of precision medicine [156]. Similarly, increasing expression levels of key genes via gene replacement therapy is another possibility [157]. Regaining normal gene function could help restore neuronal migration in MDS, potentially reducing or preventing the condition. Although these therapies hold promises, there are significant challenges to overcome, and it is not clear if they would be effective post-birth.

Future research should consider the roles of non-coding genes, including microRNAs (miRNAs) and long non-coding RNAs (lncRNAs) encoded in the MDS locus (Figure 1), which could play a pivotal role in disease progression. Non-coding RNAs have emerged as critical regulators in gene expression. For example, miR132 and miR212 are co-expressed and share similar functionalities in the brain, such as neuronal development, synaptic plasticity, and neuroprotection [158,159]. These two miRNAs are often downregulated in different neurological diseases like epilepsy, Alzheimer’s, Parkinson’s, and Huntington’s diseases [160]. This dysregulated expression of miR-132/212 could serve as a valuable diagnostic tool and an innovative therapeutic approach, leveraging its protective functions for potential benefits. Similarly, elevated levels of miR22 have demonstrated neuroprotective effects in Huntington’s disease, highlighting the potential for manipulating miR22 in vivo as a feasible therapeutic approach [161]. Therapeutically, miRNA-based strategies could involve the inhibition of specific miRNAs to correct gene expression levels in the context of MDS. This could help mitigate the effects of gene deletions, especially in 17p13.3 or other key targets outside of the MDS locus, such as DCX.

## 6. Concluding Remarks

The effective treatment of MDS requires early genetic testing, such as chromosomal microarray or DNA sequencing to identify genetic abnormalities in/near 17p13.3 and the related phenotypes, such as LIS, growth retardation, or organ dysfunction. Currently, there is no cure for MDS, with management primarily focused on controlling seizures. Despite the advances in understanding the clinical features of MDS, many aspects of MDS remain poorly understood at the molecular level. Research has identified deletions within the 17p13.3 region that contribute to a smooth brain, but there is still much to uncover regarding the complete spectrum of MDS-related genes and most especially non-coding genes. To fully understand MDS, more MDS patient samples and model systems need to be examined, particularly by applying a multi-omics approach. While transcriptomics and proteomics analyses have been performed, additional approaches like epigenomics, epitranscriptomics (e.g., m^6^A-seq), and metabolomics could provide insights into possible post-transcriptional and post-translational modifications, as well as other metabolic changes that could impact the disease state, which can then be integrated with neuronal migration and other altered pathways, such as SAM/SAH pathways, mTOR signaling, calcium signaling, and the STAT3 pathway, and possibly using artificial intelligence.

Future research needs to explore the identified molecular pathways more comprehensively in order to develop targeted treatments that address the root causes of the disease, rather than focusing solely on symptom management. Targeted therapies that suppress the STAT3 pathway (e.g., JAK inhibitors), enhance protein translation (e.g., NGF and EGF treatments), modulate the activity of the mTOR pathway (e.g., NV-5138, everolimus), and restore the SAM/SAH methylation balance (e.g., betaine treatment) hold significant promise for alleviating multiple symptoms of MDS. These strategies, though promising, require further investigation to validate their efficacy. Ultimately, the discovery of drugs that effectively target these pathways and mitigate the phenotypic manifestations of MDS also have broader applicability to other forms of lissencephaly and neurodevelopmental disorders, given their common molecular pathways involved in neuronal migration defects and brain malformations.

## Appendix A

**Table A1:** Clinical manifestations of MDS patients exhibiting a range of complications to date.

Case Representation (Additional Notes About Patient)	Age at Diagnosis and Method	Region Deleted	MDS Phenotype/Symptoms Reported	Treatment to Control the Complications
Born weighing 2.69 kg (5.93 lbs.) [20]	5 weeks old using, ultrasound and CT scan	17p13.3q25.3 deletion	Microcephaly, micrognathia, low set ears, thin upper lips, bilateral clinodactyly and cryptorchidism	Not reported
Born weighing 2.308 kg (5.08 lbs.) [70]	38 weeks of gestation, using FISH, brain ultrasound, CT scan, MRI	17p13.2 and *LIS1* haploinsufficiency in 17p13.3	Facial dysmorphism, intrauterine growth restriction, paucity of gyral and sulcal development, growth retardation, developmental delay, and seizures.	Not reported
Born weighing 975 g (or 2.15 lbs.) [133]	30 weeks of gestation, using CGH	17p13.3-p13.2 region deletion (including *PAFAH1B1* and *YWHAE*)	Seizures with apnea and behavioral arrest, epileptic spasms, severe LIS on Day 76 MRI scan	Phenobarbital administration for seizure control, corpus callosotomy (CC) to treat seizures
Presented with developmental delays and febrile-induced epileptic seizures [162]	32 months, using MRI, whole genome sequencing and video electroencephalogram	17p13.3p13.2 heterozygous inversion spanning 1.02 Mbps	A low hairline at the back, binocular esotropia, widely spaced eyes, a flat nasal bridge, broad gaps between teeth, excessive drooling, delayed psychomotor development, and mild muscle weakness	Not reported
Recurrent seizures for 5 days, born with cyanosis and respiratory distress [37]	6 months, using head CT scan	Not reported	Facial dysmorphism, growth and developmental delays, diffuse agyria, micrognathia	Not reported
Reported with pre- and postnatal growth retardation [35]	15 years, using exome sequencing	De novo 17p13.3 deletion (including *CRK*)	Intrauterine, growth retardation	Recombinant human growth hormone therapy partially decreased the height deficiency
Reported with pre- and postnatal growth retardation [35]	11 years and 10 months, using exome sequencing	De novo 17p13.3 deletion (including *CRK*)	Intrauterine, growth retardation	Recombinant human growth hormone therapy induced the growth
History of intrauterine growth retardation, short stature, intractable epilepsy, expressive language disorder, clinodactyly, and retinal freckling [38]	5 years, using aCGH and cytogenetic analysis	17p13.3 deletion (1759 kbp region including *YWHAE* and *CRK* but not *PAFAH1B1*) and a mosaic r17	Developmental delays, dysmorphic facial features, other physical abnormalities	Not reported
History of developmental delay and recurrent seizures [46]	3 years, using brain MRI and electroencephalogram	LIS/Subcortical band heterotopia (LIS/SBH) spectrum (deletion not reported)	Delayed motor coordination, pneumonia, acute gastroenteritis, mild ventriculomegaly	Carbamazepine and folic acid to control the focal seizures
Diagnosis of intra-uterine growth retardation with fetal distress and no fetal movements [32]	39 weeks of gestation, using CT scan and chromosomal analysis	Microdeletion of the terminal end of 17p	Epileptic fits and hypotonia	Fits controlled through medication
Born weighing 2.52 kg (5.6 lbs.), admitted at 6 weeks of age due to jaundice [51]	6 weeks, using SNP microarray	De novo 17p13.3 deletion (1.4 Mbp region including *YWHAE* and *CRK* but not *PAFAH1B1*)	Micrognathia, tented upper lip, broad forehead, one febrile seizure, developmental delay	Not reported
Born at 37 weeks of pregnancy with birth weight of 1844 g (or 4 lbs.). Admitted for low birth weight and respiratory distress [163]	At birth, using electroencephalography, brain MRI, and FISH	Not reported	Microcephaly, a narrow forehead, small nose and chin, and cardiac malformations, repeated afebrile seizures	Zonisamide and levetiracetam
Presented with a worsening of seizures in the setting of a Pseudomonal and Enterococcal urinary tract infection [42]	6 months, using MRI of neuraxis	*LIS1* 17p13.3 deletion	*Pseudomonal* and *Enterococcal* urinary tract infection, seizures, epilepsy, cerebral palsy, cognitive delays, prominent forehead, upturned short nose	Not reported
Born weighing 2.069 kg (4.56 lbs.) with a complicated pregnancy due to intrauterine growth retardation, delivery induced to prevent complications [41]	33 weeks of gestation, using microarray analysis and later with CT scan, ultrasound and MRI	Sub telomeric region deletion of 17p13.3 (including *YWHAE*) (see MDS1 in Figure 1)	Developmental delays, macrocephaly, ventriculomegaly, generalized seizures, idiopathic generalized epilepsy	Not reported
Prenatal diagnosis of omphalocele with mild ventriculomegaly [49]	3 years, using FISH, fetal echocardiography, MRI	17p13.3 deletion	Developmental delays, seizures, no gyral formations	Not reported
Born weighing 3.2 kgs (7.05 lbs.) and presented for growth evaluation at the age of 10.8 years [67]	10.8 years, using aCGH, MRI	17p13.3 deletion (284 kbp including *CRK* and *MYO1C*) (see MDS2 in Figure 1)	Intellectual disability, facial and limb abnormalities, feeding difficulties, delayed psychomotor development	Substantial catch-up response following growth hormone treatment
Born weighing 2.4 kg (5.3 lbs.) and presented with ocular malformation and speech delay [36]	37 weeks of gestation, using aCGH, brain MRI	Distal deletion in 17p13.3 region (554 kbps) (see MDS3 in Figure 1)	Intra-uterine growth retardation, short nose, a pointed chin and an everted inferior lip, low set ears, prominent forehead, no motor delay	Not reported
Born weighing 1.8 kg (4 lbs.); brought in due to epilepsy and developmental delays [51]	9 years, using microarray, brain MRI	17p13.3 deletion (140 kbp including *YWHAE*, *TRARG1* and *BHLHA9*, but not *CRK* or *PAFAH1B1*)	Prematurity, respiratory distress syndrome, flat mid face, hyperbilirubinemia, seizures	Not reported
Born weighing 2.8 kgs (6.2 lbs.); brought in due to new-onset infantile spasms and a history of delay [28]	5 months, using MRI, electroencephalogram	17p13.3 deletion (contiguous large heterozygous deletion including *PAFAH1B1*)	Prominent forehead, cardiac defect, bitemporal hollowing, short nose with upturned nares, mild hypotonia, thickened upper lip, pachygyria	On oral vigabatrin and neurodevelopmental therapy until 9 months old
Grade I LIS with midline calcification and aspiration pneumonia [93]	18 months, using head ultrasound, G-band chromosome analysis, and electroencephalogram	17p13.3 deletion	Dilated bilateral ventricles, prominent forehead, bitemporal hollowing, short nose with upturned nares, prominent upper lip, micrognathia	Not reported
Worsening abnormal movements beginning from 3 months of age [45]	4 months, using whole exome sequencing and electroencephalogram	Not reported	Developmental delays	Intravenous pyridoxine administration
Admitted with increased seizures, urinary tract infection, and a buttock abscess [42]	6 months, using MRI	17p13.3 deletion	Agyria, ventriculomegaly, dermal sinus tract	Not reported
Born weighing 4 pounds 9 ounces, with pregnancy complications due to intrauterine growth retardation [41]	38 weeks of gestation, using FISH	Terminal deletion of 17p13.3 distal to MDS locus	Ventriculomegaly, epileptiform discharges, developmental delays, hypotonia, frontal bossing, hypertelorism	Not reported
